# The relationships between parents’ and children’s screen times on body mass index: a cross-sectional path analysis

**DOI:** 10.1186/s12889-022-14664-x

**Published:** 2022-11-28

**Authors:** Kaori Ishii, Ai Shibata, Mohammad Javad Koohsari, Koichiro Oka

**Affiliations:** 1 Faculty of Sport Sciences, Waseda University, 2-579-15 Mikajima, Tokorozawa, 359-1192 Japan; 2 Faculty of Health and Sport Sciences, University of Tsukuba, Tsukuba, Japan; 3 School of Knowledge Science, Japan Advanced Institute of Science and Technology, Nomi, Japan

**Keywords:** Sedentary behavior, Elementary school children, Sitting, Body mass index, Asia

## Abstract

**Background:**

Understanding factors contributing to an individual reducing screen time is essential for promoting a healthy weight. Parents’ behavior affects children by influencing their daily decision-making through modeling, rules or restrictions, social support, and co-participation. We examined how the direct and indirect effects of parents’ and children’s behaviors regarding screen time influenced body mass index (BMI) among Japanese elementary school children.

**Methods:**

We included 283 Japanese children, one child per household, aged 6–12 years, who were randomly selected from resident registries of two cities. The questionnaires were completed by children and their mothers and fathers. Screen time and sociodemographic attributes, including sex, age, employment status, height, and weight, were assessed using a mail-based survey. Path analyses were conducted to determine associations among children’s, fathers’, and mothers’ variables. It was hypothesized that after controlling for household income and children’s sex and age, mothers’ and fathers’ screen time on weekdays and weekends would be related to children’s weekdays and weekend screen time, respectively. In addition, we hypothesized that children’s weekday and weekend screen time was related to children’s BMI.

**Results:**

Both fathers’ and mothers’ weekday screen times were associated with children’s weekday and weekend screen times. BMI was affected by children’s weekday screen time (0.117). The path coefficients for the indirect effects of mothers’ and fathers’ screen time on children’s BMI through children’s weekday screen time were 0.016 from the fathers’ weekday screen time and 0.024 from the mothers’ weekday screen time (GFI = .980, AGFI = .953, RMSEA = .030, AIC = 93.030).

**Conclusions:**

Both fathers’ and mothers’ weekday screen times indirectly affected children’s BMI through children’s weekday screen time among Japanese elementary school children. The strongest indirect effects could be seen by examining the paths of a mother’s weekday screen time through children’s screen time to BMI. Mothers who spend much time with their children are role models, and their behavior could affect the child’s behavior. The findings imply that intervention strategies to reduce screen time in children should also focus on modeling the mothers’ behavior.

## Background

Obesity in children is a public concern worldwide and is associated with type 2 diabetes, hypertension, and an increased risk of obesity in adulthood [1, 2]. For example, in Japanese school-aged children, 11.1% of boys and 8.8% of girls aged 11 years were classified as obese in 2019 [3]. Compared to other developed countries, levels of obesity in Japanese school-aged children are low [4]; however, the percentage has grown in the last 10 years [3]. Especially in girls, elementary school-age students are more likely to be obese or overweight than junior high school or high school-age students [3]. Therefore, preventing obesity in children is vital for their future health.

Excessive sedentary behavior is associated with poor health and can result in increased adiposity, worse cardiometabolic health and fitness, impaired behavioral conduct/pro-social behavior, and reduced sleep duration [5]. For children, several current physical activity guidelines [6, 7] recommend recreational screen time of no more than 2 h per day (i.e., watching television [T.V.], digital video discs, or videos, playing T.V. games, or using computers or the internet) and avoiding prolonged periods of sitting. Nevertheless, children spend too much time on their recreational screen time worldwide [8]. For instance, in the United States, 66% of children spend at least 2 h of screen time per day [9]. In Japan, approximately 60% of children have been found to exceed the 2 h per day mark of screen time [10].

Parents play an essential role in children’s daily decision-making through modeling, rules or restrictions, social support, and co-participation [11, 12]. Previous review studies have shown that parents’ screen time is positively correlated with children’s screen time [13–27], and co-viewing with parents has been associated with increased screen time in children [28, 29]. Moreover, the impact on children’s screen time appears to be dependent on the sex of the guardian, as a previous study reported that mothers’ screen-based behaviors showed a positive correlation with children’s screen time [17, 28, 29]. However, few studies have considered gender differences in parental roles. Studies that have examined both the father’s and mother’s influence on children’s sedentary behavior report that compared to the father’s sedentary behavior, the mother’s sedentary behavior influences the child’s sedentary behavior more [28, 29]. Xu et al. [30] concluded that reducing parents’ screen time could decrease their child’s screen time. Therefore, examining the impact of both fathers’ and mothers’ screen time on children is necessary.

In addition to the influence of the parents’ gender, it has been reported that the influence of the parents’ screen time on children’s screen time varies between weekdays and weekends [19, 27]. Jago et al. (2014) [27] concluded that associations observed between parent and child screen-viewing were different between weekdays and the weekend; they showed that on a weekday, children were 3.4 times more likely to exceed 2 h of screen viewing if their father watched T.V. for at least 2 h per day, while for a weekend day, children were 4.8 times more likely. There were similar associations for mothers; children were 3.7 times more likely to exceed 2 h of screen viewing if their mother watched T.V. for at least 2 h per day on a weekday, while children were 4.7 times more likely for a weekend. However, to our knowledge, only a few studies have examined the differentiation between weekdays and weekends [18, 19, 27].

The indirect effects and the strength of paternal and maternal screen time on children’s screen time and body mass index (BMI) have not been examined. However, some studies have examined each of these variables directly, such as parents’ screen time and children’s screen time [13–30] or children’s screen time and BMI [5]. Considering the impact of the behaviors of both father and mother on children in real life, parental behaviors may impact children’s screen time and BMI, and suggestions for specific interventions to improve children’s health might be possible through research. Thus, the present study examined how the direct and indirect effects of parents’ and children’s screen time behaviors influenced children’s BMI among Japanese elementary school children.

## Methods

### Participants and data collection

**Fig. 1:**
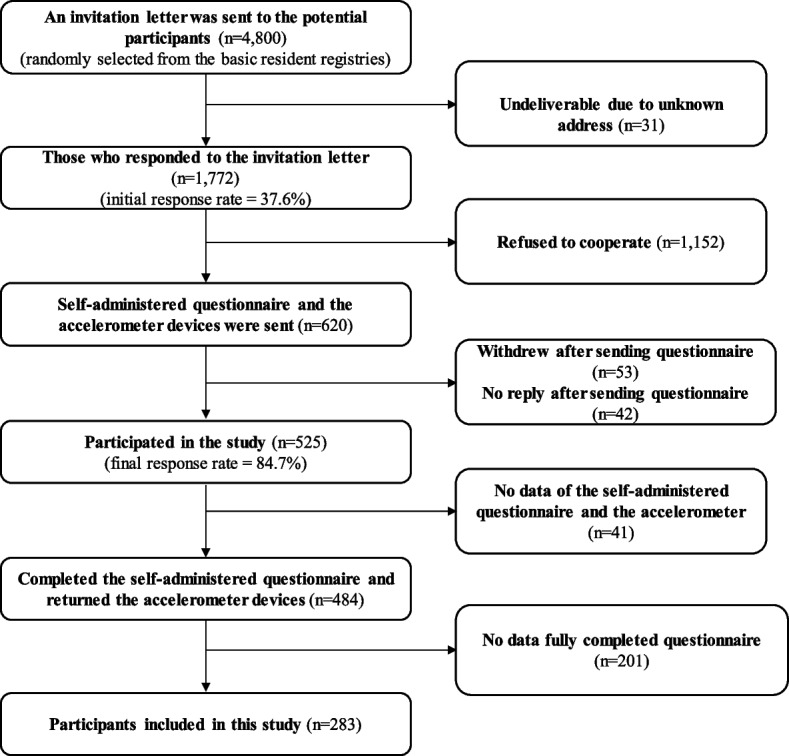
Participation flow

The present cross-sectional study was conducted in a cohort of children living in Musashino City, Japan, in 2018 and Kokubunji City, Japan, in 2017. A total of 4800 potential residents aged 6–12 years, one child per household, were randomly selected from the residential registries of their respective cities. These selected children and their parents were the study participants. Musashino (population: 150,660 in October 2021) and Kokubunji (population: 130,636 in October 2021) are cities in Tokyo, Japan. Because both cities have approximately the same size of age population, the present study stratified them to account for this population ratio. Potential participants were stratified by sex (boys/girls) and school grade (1st grade: 6–7 years, 2nd grade: 7–8 years, 3rd grade: 8–9 years, 4th grade: 9–10 years, 5th grade: 10–11 years, and 6th grade: 11–12 years). All mailings related to the mail-based survey were addressed to the children and their parents. First, invitation letters explaining the study were sent to all potential participants. A questionnaire and accelerometer were sent to those who responded to the invitation letter indicating they were willing to participate. To encourage a response, potential participants were told that a 1000-yen book voucher would be offered to those who returned the questionnaire and accelerometer. Non-respondents were sent one reminder about the responses to a questionnaire and accelerometer. A total of 1772 families (37.6% overall response rate; 881 responders from Musashino and 891 from Kokubunji) responded to the invitation. Then, self-administered questionnaires for children and their mothers and fathers were given, which included questions about sociodemographic variables, screen time, and children’s height and weight, were mailed to those who responded that they would be willing to participate (620 individuals; 12.9% of all the invited respondents who mentioned they would be willing to participate, 310 people in each city). A total of 484 tryads completed the questionnaire and accelerometer measurements (78.1% overall response rate; 81.0% from Musashino, and 71.3% from Kokubunji). Data from 283 children and their parents who fully completed both questionnaires were included in the analysis (Fig. 1). Participation was voluntary, and confidentiality was ensured. A previous study [31] suggested that children younger than 10 years cannot report their activity patterns accurately or reliably. Alternatively, parental reports of physical activity among 6-year-olds have been shown to strongly correlate with heart rate measures during physical activity [32]. Therefore, parents of the children were asked to complete the questionnaire with their children. In addition, only mothers were asked to respond to the mothers’ questionnaire and only fathers to the fathers’ questionnaire.

### Standard protocol approvals, registrations, and patient consent

All children, mothers, and fathers signed an informed consent form before answering the questionnaire. The Ethics Committee of Waseda University, Japan, approved the study before its commencement (2017–245). The present study was conducted in accordance with the principles of the 2013 Declaration of Helsinki.

### Measures

#### Self-reported screen time

Domain-specific sedentary behaviors were assessed using a questionnaire. For children, sedentary behavior was divided into six domains [10] (1) reading or listening to music, (2) T.V. or video viewing, (3) T.V. game use, (4) internet or e-mail (computer or tablet) use outside of class, (5) doing homework or assignments, and (6) car travel for transport. Participants were asked how many days on average per week and how much time (hours and minutes) on average per day they engaged in these sedentary behaviors during weekdays and weekends in each domain. Then the weekly frequency was multiplied by the number of minutes per day. Each domain-specific sedentary behavior was examined separately, and we calculated the average total number of minutes for each school week (Monday–Friday) and weekends (days × minutes per day). Screen time was calculated using the total of domains (2), (3), and (4). The mothers and fathers were asked to report daily average sedentary time (hours and minutes) over the past 7 days, separately for workdays (weekdays for non-employed) and non-workdays (weekend for non-employed) across the following six domains: (1) being transported to and from a place by car; (2) using public transport; (3) at work; (4) watching television, videos, and DVDs; (5) using a computer, cell phone, and tablet P.C. outside of working hours; and (6) during leisure time (excluding watching television, videos, and DVDs) [33]. The total minutes of daily average screen time was calculated by summing (4) and (5) separately for workdays and non-workdays. Average daily values of sedentary time were calculated with weighting to account for the number of weekdays and weekend days.

#### Sociodemographic factors

Data on children’s age and sex were collected from the residential registries. The children’s current weights and heights were obtained from the questionnaire for children’s responses. BMI was calculated from the height and weight data (BMI = weight/height [2]). Children’s BMI percentiles were calculated using the metric system of the Centers for Disease Control and Prevention [34]. Children were classified according to the recommended BMI-for-age cutoffs [34]: < 5th percentile, underweight; 5–85th percentile, normal BMI; and ≥ 85th percentile, overweight or obese. Additionally, household income level per year (< 3, ≥3–< 5, ≥5–< 7, ≥7–< 10, or ≥ 10 million yen) and employment status (employed or not) were assessed from the parents’ responses.

### Statistical analyses

The data analysis involved assessing the replies from the 283 children, fathers, and mothers who had fully responded. Path analyses were conducted to determine the presence of any associations between the children’s, fathers’, and mothers’ variables. It was hypothesized that after controlling for household income and children’s sex and age, mothers’ and fathers’ screen time during the weekdays and the weekend would be related to children’s screen time during the weekdays and the weekend, respectively; it was also hypothesized that children’s weekday and weekend screen time would be related to BMI. Path coefficients and correlations are reported as standardized estimates. The model was assessed using the goodness-of-fit statistic (GFI), adjusted goodness-of-fit statistic (AGFI), root mean square error of approximation (RMSEA), and Akaike information criterion (AIC). GFI and AGFI indices were used to measure how well the model fit the data. Values of 0.90 or greater indicated a good model fit [35]. RMSEA is a measure of the descriptive measures of overall model fit. An RMSEA score value lower than 0.05 indicated a good fit [36]. A lower AIC value for a model indicated a better fit than the other models [37]. A model was considered to fit the data well when the following criteria were met: GFI > 0.90, AGFI > 0.90, RMSEA < 0.06, and a lower AIC value compared with competing models. Statistical significance was set at *p* < 0.05. The data were analyzed using path analyses estimated using IBM SPSS AMOS 27.0 J for Windows (IBM Corp., Armonk, N.Y., USA).

## Results

### Demographics and screen time of the participants

A total of 283 Japanese children and dyads (127 boys and 156 girls; 144 in Musashino and 139 in Kokubunji) completed the survey. Table 1 shows the demographic characteristics of the participants and their screen time during the weekdays and weekends. The children’s mean (standard deviation) age was 8.7 (1.7) years old, and 82.3% of children had a healthy weight status. Regarding socioeconomic status, 41.7% of families had a household income of ≥10,000,000 yen. The child’s, mother’s, and father’s mean screen time were 112.5 (92.1), 148.1 (112.4), 123.1 (86.6) on weekdays, and 155.4 (102.1), 167.5 (108.3), and 212.9 (134.8) on weekends, respectively. The proportion of employed mother and father respondents were 66.1 and 99.3%, respectively.

**Table 1:** Descriptive characteristics (numbers and percentages)

Variables	n	%
Overall	283	100.0
Sex		
Boys	127	44.9
Girls	156	55.1
Age, group		
6 years	18	6.4
7 years	61	21.6
8 years	69	24.4
9 years	49	17.3
10 years	38	13.4
11 years	29	10.2
12 years	19	6.7
Mean ± S.D.	8.7 ± 1.7	
BMI percentile for age and sex		
Underweight (< 5th %ile)	21	7.4
Normal BMI (5th - 85th %ile)	233	82.3
Overweight or obese (≥ 85th %ile)	29	10.2
Household income level		
< 3,000,000 yen	3	1.1
< 5,000,000 yen	26	9.2
< 7,000,000 yen	41	14.5
< 10,000,000 yen	95	33.6
≥ 10,000,000 yen	118	41.7
Employment status, employed		
Mother	79	66.1
Father	280	99.3
Screen time-weekday, min/day, Mean ± S.D.		
Children	112.5 ± 92.1	
Mothers	148.1 ± 112.4	
Fathers	123.1 ± 86.6	
Screen time-weekend, min/day, Mean ± S.D.		
Children	155.4 ± 102.1	
Mothers	167.5 ± 108.3	
Fathers	212.9 ± 134.8	

*Abbreviations*: *BMI* Body mass index, *S.D*. Standard deviation

### Direct and indirect effects of parents’ and children’s screen time behaviors on BMI

**Fig. 2:**
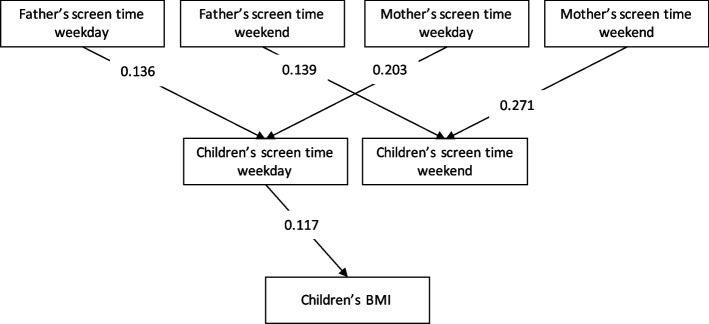
Direct and indirect effects of parents’ and child’s screen time behaviors on BMI. (goodness-of-fit statistic = 0.980, adjusted goodness-of-fit statistic = 0.953, root mean square error of approximation = 0.030) Only statistically significant paths are indicated in this figure. All paths are statistically significant at *p* < 0.05. BMI, body mass index

Figure 2 shows the direct and indirect relationships between weekday and weekend mothers’ and fathers’ screen time, children’s weekday and weekend screen time, and BMI. All path coefficients are standard partial regression coefficients. With the standard partial regression coefficients, the magnitude of each factor can be directly compared with the other factors in the model.

The present study identified no significant associations between (1) fathers’ or mothers’ weekday screen times and children’s weekend screen times, or (2) between fathers’ or mothers’ weekend screen times and children’s weekday screen times, or (3) between children’s weekend screen times and BMI (GFI = 0.985, AGFI = 0.953, RMSEA = 0.029). Recalculating the model using modified indices reduced the AIC value from 96.351 to 93.030. Thus, the final model demonstrated an acceptable fit (GFI = 0.980, AGFI = 0.953, RMSEA = 0.030). Both fathers’ and mothers’ weekday screen times were seen to affect children’s weekday screen times (from fathers’ weekday screen times: 0.136, from mothers’ weekday screen times: 0.203), and both fathers’ and mothers’ weekend screen times were seen to affect children’s weekend screen times (from fathers’ weekend screen times; 0.139, from mothers’ weekend screen times; 0.271). BMI was affected by the children’s weekday screen times (0.117). The path coefficient for the indirect effects of mothers’ and fathers’ screen times on BMI through children’s weekday screen times was 0.016 from the fathers’ weekday screen times and 0.024 from the mothers’ weekday screen times.

## Discussion

The present study indicated that both fathers’ and mothers’ weekday screen times indirectly affected children’s BMI through children’s weekday screen times among Japanese elementary school children.

There have been no consistent research findings on whether weekday or weekend screen times influence children’s BMI. Only children’s weekday (and not weekend) screen times were associated with BMI in the present study. A study of adolescents showed no significant correlation between weekday and weekend T.V. viewing times and BMI [38]; on the other hand, a study of preschool children showed that only children’s weekend screen times were associated with BMI [18]. The small number of studies evaluating the effects on BMI that separated weekday and weekend screen times may have contributed to the lack of consistent results because whether it is a weekday or a weekend, a previous study [5] suggested that the total screen time has an impact on BMI. However, to reduce children’s screen time, it would be helpful to consider what factors should be addressed on weekdays and weekends separately to adopt those concrete strategies. For example, a study of preschool children [18] reported that there was an association between screen time and BMI on weekends; however, the reason for no association between screen time and BMI on weekdays was that the total amount of time spent in front of a screen on weekdays was low. After all, the preschooler/students went to kindergarten/preschool/school. In this study, the total screen time per week (9.4 hours on weekdays, 5.2 hours on weekends) was also significantly higher on weekdays than at weekends, which may be one of the reasons for this discrepancy.

Fathers’ and mothers’ weekday screen times influenced children’s weekday screen time, which influenced BMI, and for both weekdays and weekends, the influence of a mother’s screen time was stronger than that of a father’s screen time. One previous study on the influences of parent screen time indicated that increased T.V. time of the child was associated with increased father’s T.V. time (father odds ratio = 2.33 times, mother odds ratio = 2.24 times) than with mother’s T.V. time [24]. The impact of fathers’ T.V. time on children’s T.V. time was stronger than that of mother’s T.V. time in elementary school children (mean age 7.6 years), a similar cohort to the present study [24]. On the other hand, adolescents aged 12–13 years watching T.V. ≥ 2 hours per day were associated with mothers who watched T.V. ≥ 2 hours per day, but there was no association with fathers’ T.V. time [28]. One of the reasons that these relationships were stronger for mothers than fathers in this study was that children in Japan spend more time with their mothers. A 2016 Ministry of Internal Affairs and Communications survey showed a significant difference in the average time spent with elementary school-aged (over 10 years old) children per day: 4 hour 52 min for mothers and 2 hour 40 min for fathers [39]. Moreover, this trend seems to be associated with the employment rate of both parents in this study, as fathers were 33.2% more likely than mothers to be employed. Parents are role models for their children, and by shared time, their behavior could affect a child’s behavior. Therefore, maternal modeling might have a stronger effect than paternal modeling on child behavior, as Japanese children spend much time with their mothers.

Moreover, a previous study [19] that examined weekday and weekend screen times separately found no association between parents’ and children’s screen times; this study did not examine the fathers’ and mothers’ screen times separately. In contrast, another study [27] that examined weekday and weekday screen times found associations between children’s weekday and weekend screen times and both fathers’ and mothers’ screen times on weekdays and weekends; this study examined the fathers’ and mothers’ screen times separately. In a study with at least one parent and a child aged 5–6 years, when parents exceeded 2 h of T.V. watching, children were 3.4 times and 4.8 times more likely to spend ≥2 hours T.V. watching during the weekdays and weekends, respectively, if their father exceeded the threshold; the odds were 3.7 for the weekdays and 4.7 for the weekends if their mothers exceeded the threshold [27]. This means that the influence of fathers and mothers may be different on weekdays and weekends. However, in the present study, although both fathers’ and mothers’ weekday screen times influenced children’s weekday screen times and both the fathers’ and mothers’ weekend screen times influenced children’s weekend screen times, the influence of the mothers’ screen times was stronger than that of the fathers’ screen times on both weekdays and weekends. Japanese children aged 10–14 years spend an average of 258 min with their mothers and 125 min with their fathers on weekdays, but on Sundays, they spend 411 min with their mothers and 272 min with their fathers; thus, although the children spend some time with their fathers, they spend 1.5 to 2 times longer time with their mothers [39]. Given this difference in Japan, the results of this study, which examined weekday and weekend screen times and paternal and maternal screen times separately, help develop intervention strategies to prevent obesity in Japanese children and reduce screen time.

Regarding the indirect effects on BMI, the strongest path was the influence of maternal weekday screen times on BMI via children’s weekday screen times (0.024). On the other hand, fathers’ indirect passes were 0.016. This may be partly due to the strong maternal commitment to weekday children’s screen times, which suggests that mothers’ influence should be considered when reducing screen time to improve BMI among Japanese children. Although the study did not examine fathers or mothers separately and was conducted in preschool-aged children, the indirect effect of parental screen time on children’s BMI was found only on weekends [18]. In addition, the study that did not examine weekdays and weekends separately examined fathers’ and mothers’ physical activities separately and showed that the effect of mothers’ physical activity on children’s physical activity, which affects children’s BMI, was significant, but the effect of fathers’ physical activity was not [22]. There is a lack of research on modeling fathers’ and mothers’ behaviors on children’s behaviors, and of children’s behaviors on BMI; further research should be conducted in the future. For example, previous studies have reported efforts to reduce sedentary behavior in children and their parents through interventions targeting mothers and children [40]. There have been efforts to reduce parental sedentary behavior through parent education [41]. In the case of Japanese children, these interventions have not been reported. These efforts are expected to improve maternal literacy and reduce children’s screen time, thereby improving their anthropometric indices.

Some limitations of this study should be considered. First, the study’s cross-sectional nature limits the conclusions that can be drawn about the cause and effect of the observed relationships between parents’ and children’s screen times and anthropometric factors. Second, to estimate screen time, the study relied on self-reported measures with the potential for error owing to different interpretations of the questions. However, for children, the screen time scale has been used in a national survey of Japanese elementary school children [42], and the parent screen time scale confirmed its reliability and validity [33]. Third, the study respondents were slightly different from the general population. To estimate the representativeness of the participants’ responses, the population percentage by age group in the present study was compared with data from population estimates [43]. The proportion of boys and girls aged 6–12 years was 45% (Kokubunji;44.6%, Musashino;45.1%) for boys and about 55% (Kokubunji;55.4%, Musashino;54.9%) for girls in this study, while the national data was about 51% for boys and 49% for girls. Therefore, the essential characteristics of respondents might have been biased. The findings in such a setting may not sufficiently apply to the general population. However, the present study randomly selected participants from a registry of each city’s residential addresses, allowing an equal number of potential selects to be obtained from both sexes and each age group category between 1st grade and 6th grade. Although only sex and age distributions, the comparable variables investigated in this study, are compared, the present study population can be considered to have characteristics of the general population. Despite these limitations, few studies have been conducted on this topic in a randomly selected Japanese population, and the findings from the present study will contribute to a greater understanding of parental influences on children’s screen time and anthropometric factors and may also help to develop new strategies and interventions to promote public health and well-being in Japan.

## Conclusions

The present study indicates that both the fathers’ and mothers’ weekday screen times indirectly affected children’s BMI through children’s weekday screen times among Japanese elementary school children. The influence of the mothers’ screen times was stronger than that of the fathers’ screen times. The strongest indirect effects were seen by examining the paths of the mothers’ weekday screen times via children’s screen times to BMI. The total effect of the mothers’ weekday screen times on BMI was 0.024. The present study’s findings imply that intervention strategies to reduce screen time should also focus on mothers’ modeling of children’s health status.

## Data Availability

The datasets generated and/or analyzed during the current study are not publicly available because ethical considerations but are available from the corresponding author on reasonable request.

## Abbreviations

AGFI: Adjusted goodness-of-fit statistic
AIC: Akaike information criterion
BMI: Body mass index
GFI: Goodness-of-fit statistic
RMSEA: Root mean square error of approximation
T.V.: Television
